# Stochastic finite-fault method controlled by the fault rupture process

**DOI:** 10.1016/j.mex.2020.100798

**Published:** 2020-01-25

**Authors:** Hong Zhou, Ying Chang

**Affiliations:** a Institute of Geophysics, Cna Earthquake Administration, Beijing, 100081, China; b Department of Earth and Space Sciences, Southern University of Science and Technology, Shenzhen, Guangdong, 518055, China

**Keywords:** The stochastic finite-fault method controlled by the fault rupture process, NNSIM, Non-uniform stress drop distribution, Non-uniform time function

## Abstract

We present an improved stochastic finite-fault method controlled by the fault rupture process (NNSIM) which can make the simulated strong ground motion at a broad frequency band similar to real ground motion of the Ms 7.0 Lushan earthquake by introducing the fault physical rupture process into the stochastic finite-fault method. Two obvious improvements are obtained: 1) the non-uniform time window functions produce various shape of the simulated time series instead of one single spindle shape; 2) the non-uniform stress drops and the non-uniform time window functions improve obviously simulated pseudo-spectral acceleration (PSA), especially, the low-frequency part.

• We present an improved stochastic finite-fault method controlled by the fault rupture process (NNSIM) which can make the simulated strong ground motion at a broad frequency band similar to real ground motion by introducing the fault physical rupture process into the stochastic finite-fault method. Its validity was tested by the comparisons with records of Lushan earthquake.
• The non-uniform time window functions produce various shape of the simulated time series instead of one single spindle shape.
• The non-uniform stress drops and the non-uniform time window functions improve obviously simulated pseudo-spectral acceleration (PSA), especially, the low-frequency part.

## Graphical abstract

**Figure:**
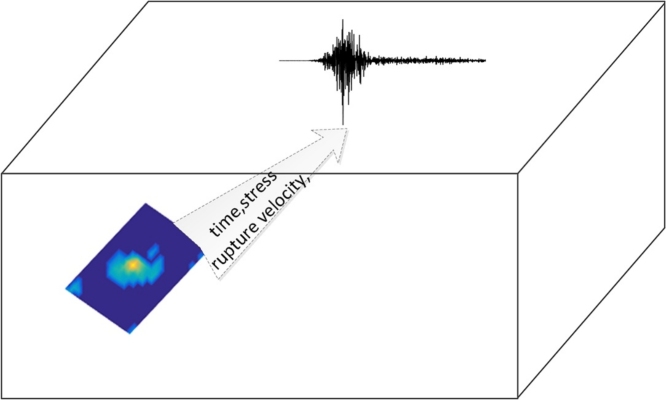

**Specification Table**

**Table:** 

Subject Area:	• Earth and Planetary Sciences • Engineering
More specific subject area:	The ground-motion simulation
Method name:	The stochastic finite-fault method controlled by the fault rupture process
Name and reference of original method	Hong Zhou, Ying Chang. Stochastic finite-fault method controlled by the fault rupture process and its application to the Ms 7.0 Lushan Earthquake. Soil Dynamics and Earthquake Engineering, 2019, 126, https://doi.org/10.1016/j.soildyn.2019.105782
Resource availability:	If applicable, include links to resources necessary to reproduce the method (e.g. data, software, hardware, reagent)

## Method details

### The implementation of the method

Stochastic finite-fault methods are effective ground-motion time-series simulation methods. The rupture process of a large fault has a non-uniform slip, a non-uniform stress drop, a non-uniform rupture velocity, and a non-uniform source time function at different points in the fault. Its implement in the stochastic finite-fault method makes the simulated strong ground motion hold the specific physical meaning and reproduce the real ground motion. Thus the new method of non-uniform stochastic finite-fault method (abbreviated as NNSIM) causes two main modifications: 1) Replace the predetermined time function with different source time functions for each subfault to improve low-frequency results including the envelopes of accelerations and velocities. 2) Replace the uniform stress drop with a distribution of non-uniform stress drops to reduce the dependence of simulated results on a single stress drop. In order to simulate the ground motion some key parameters need to be obtained as follows:

1) Time window functions

For one actual earthquake, the fault rupture process including low-frequency slip rate time function, seismic moment on the finite fault model can be inversed based on seismic records. The slip rate time functions with different time history shapes are normalized, then used as the time window functions substituting the Saragoni-Hart function for simulating the ground motion. The normalized slip rate time functions containing the low frequency characters of the source such as multiple rupture peaks make the shape of the simulated time series more similar to real ground motion.

1) Stress drop/ Stress parameter

Stress drop/ Stress parameter Δσ is a very important parameter affecting the frequency and the magnitude of the simulated ground motion [1]. Based on the fault rupture process, the non-uniform stress drop distribution can be calculated according to Ripperger and Mai [2]. Substituting uniform stress drop with the non-uniform stress drop distribution into the stochastic finite-fault method makes the simulated results agree to real records better.

Besides the above two main source parameters, the non-uniform slip rate time functions also provide various rupture delays and different seismic moment release in the finite fault. The various rupture delays reflect the change of the rupture velocities. The rupture delay can be obtained by the first-arrival picking technique. After the determination of all the source parameters, the ground motion can be calculated following the steps of the pre-existing stochastic finite-fault method [1].

## Method validation

**Fig. 1:**
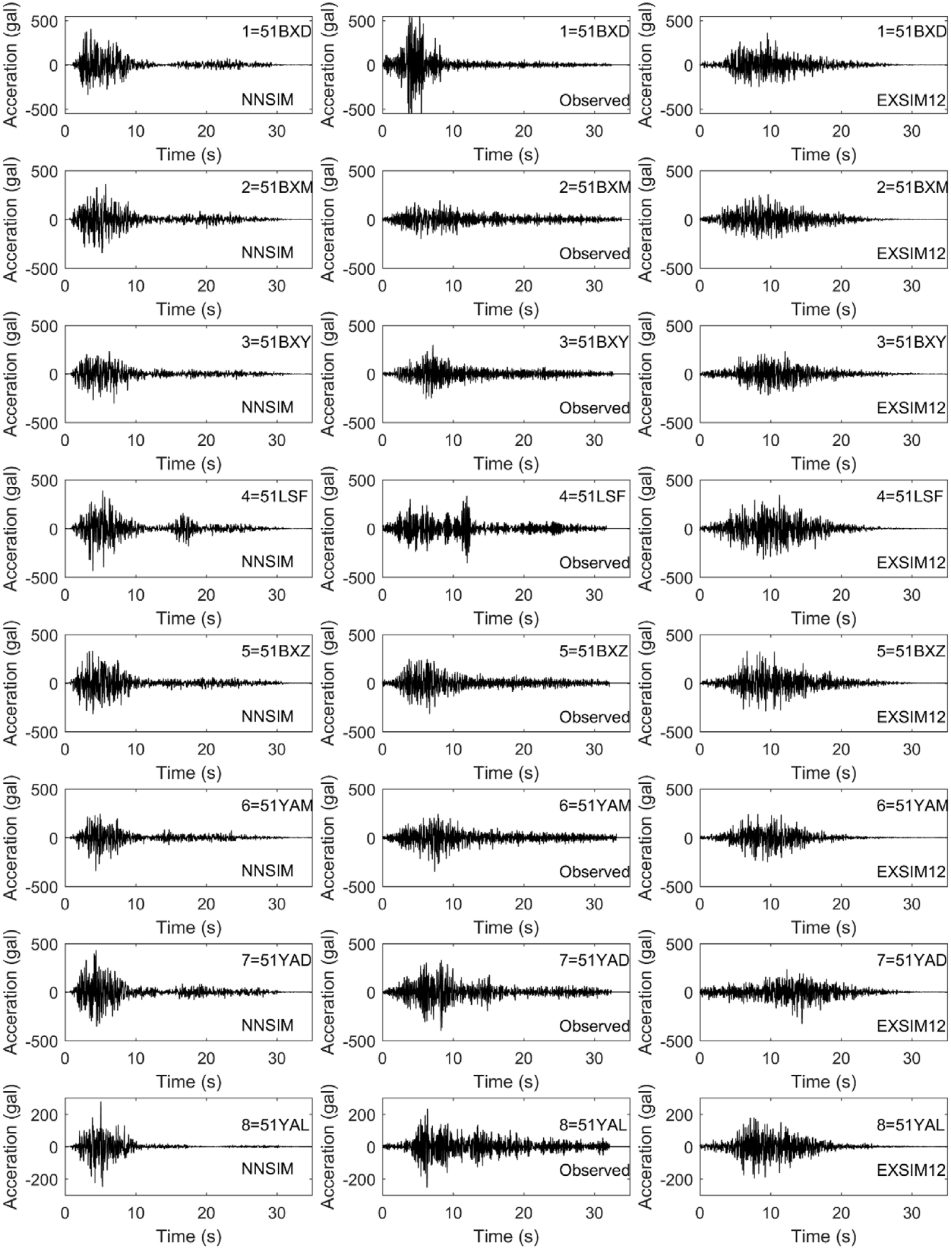
Comparison of horizontal accelerations. The left column shows the calculated results using NNSIM, the middle column shows the observed records, and the right column indicates the simulation results from EXSIM12.

**Fig. 2:**
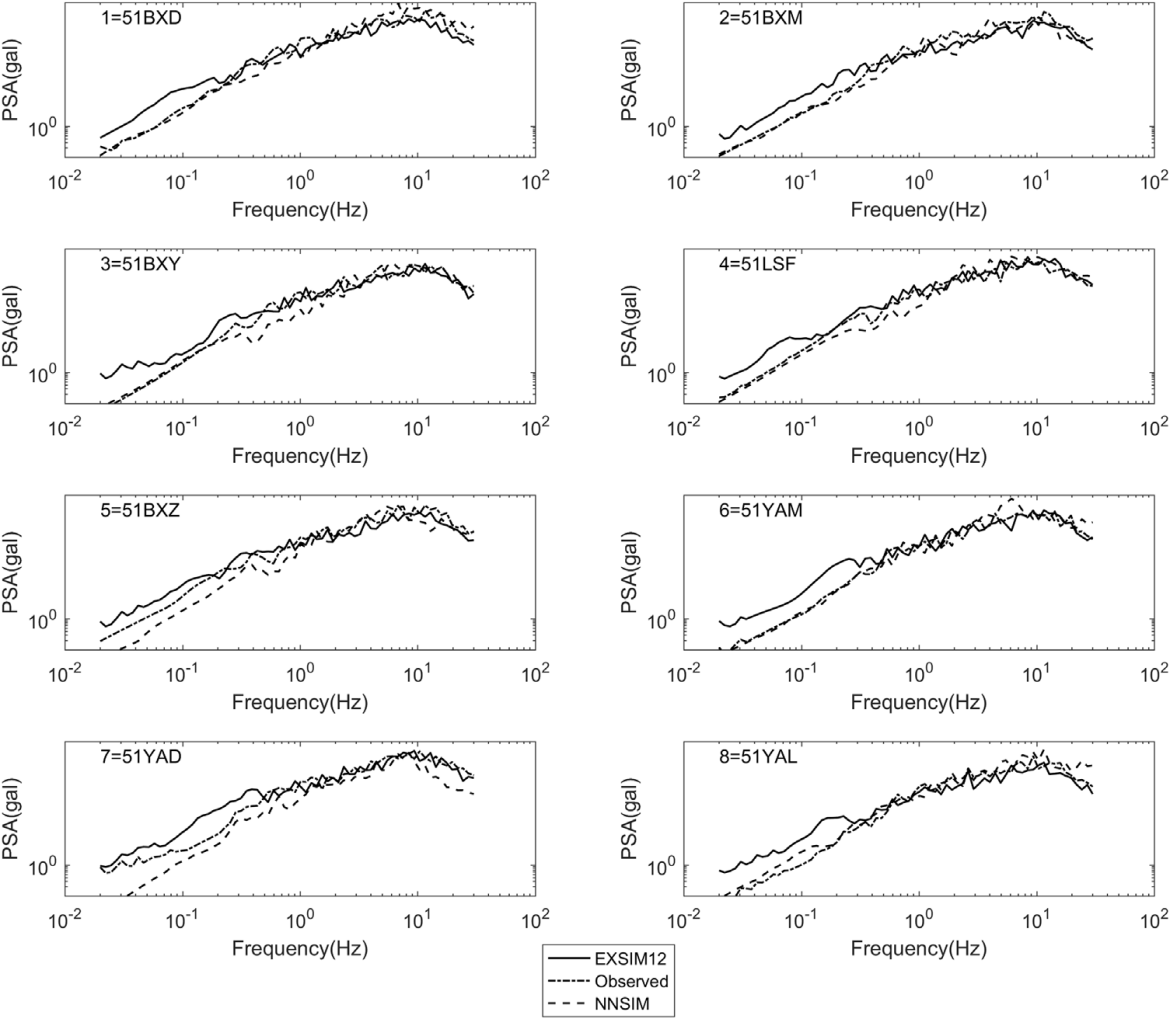
Comparison of PSA due to the NNSIM results, EXSIM12 results, and observed records.

On April 20, 2013, an Ms 7.0 earthquake struck Lushan County in China at 8:02 am Beijing time. We obtain source parameters, including non-uniform stress drop distribution, non-uniform slip rate time function, non-uniform rupture velocity and non-uniform seismic moment, based on Zhang et al.’s inversion results using teleseismic data. Combining these source parameters with stochastic finite-fault methods, we simulate the ground motions of the Ms 7.0 Lushan Earthquake, which are compared with observed records and results simulated by stochastic finite-fault methods (Version of EXSIM12). Fig. 1, Fig. 2 respectively shows the comparisons of the acceleration time histories and the pseudo-spectral acceleration (PSA), which show NNSIM can provide results more similar to observed records.

## Declaration of Competing Interest

The authors declare that they have no known competing financial interests or personal relationships that could have appeared to influence the work reported in this paper.
